# Improving the patient's experience

**Published:** 2008-12

**Authors:** C Patricia Fathers, Sue Stevens

**Affiliations:** Senior Lecturer, Health Promotion, School of Health and Social Sciences, Middlesex University, Hendon Campus, The Burroughs, London NW4 4BT, UK.; Nurse Advisor, *Community Eye Health Journal*, International Centre for Eye Health, London School of Hygiene and Tropical Medicine, Keppel Street, London WC1E 7HT, UK.

**Figure:**
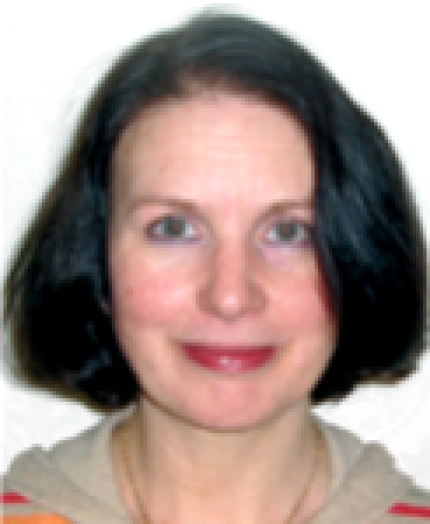


**Figure:**
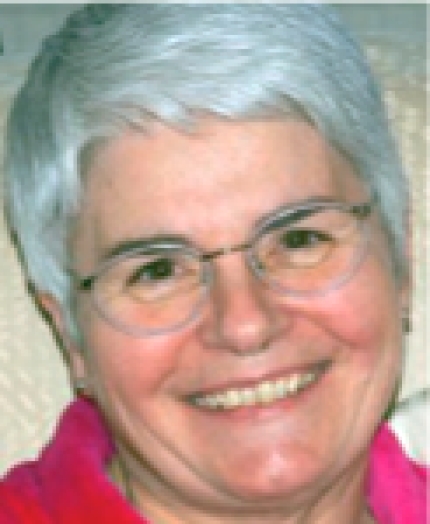


When arriving at the eye care unit, patients often feel unsure of what is going to happen, anxious, and vulnerable. Many have never found themselves in a hospital setting before or have never travelled or slept away from home.

It is an integral part of eye care to make sure a patient's experience is a positive one. This article offers suggestions for good, evidence-based, practice to improve this experience.

Our suggestions should necessarily be adapted to local context: resource-poor settings are particularly challenging work environments and staff may need to display more ingenuity in working towards good practice, when striving to achieve the goals of VISION 2020.

## Communication

Good communication is of greatest importance in all the caring professions. It is crucial at every level — between disciplines, and between staff and patients and their families.

If the eye care team is able to inform patients, instil confidence in them, and convince them of the need for treatment or follow-up, this can actually make the difference between successful and unsuccessful outcomes.^1^ It is always important to consider the patient's point of view.

### Patient information

Accessible and correct information is the key to good communication with the patient and his/her family.

**Figure:**
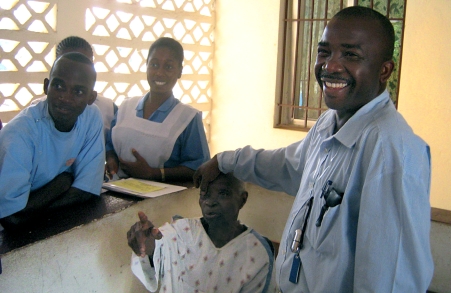
Being friendly and approachable facilitates communication with patients. LIBERIA

**Verbal information:**

- Speak in a clear and friendly manner, and avoid using medical jargon.
- The patient should not feel rushed; adapt your pace, particularly if he/she has another sensory deficit (e.g. deafness).
- You will communicate more effectively if you are friendly and approachable, rather than ‘business-like’.
- At appropriate moments in the conversation, you can check if the patient has understood the information, by asking questions such as: “Can you tell me the date and time of your next clinic appointment?”. You should also ask if the patient has any unanswered questions.
- Consider potential language barriers. Interpreters can facilitate a stress-free interaction. If language has been a problem, you should make a note of it in the patient's records. This helps to plan ahead and to make sure you include the appropriate interpreter for future appointments.

**Written information:**

Effective written information should help patients and their families to understand and remember a discussion, and it should contain all the important points.

A permanent written record of the information also offers another advantage; it gives all members of staff a standard set of information points that they should remember to include in their discussions with patients and families.^2^

**Figure:**
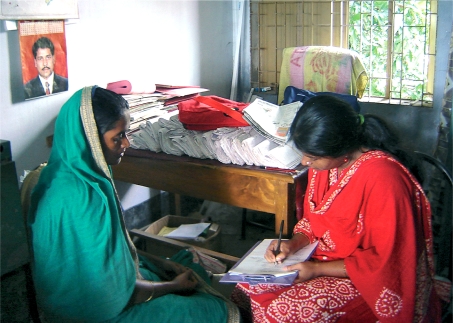
It is important to listen to the patient's point of view. BANGLADESH

For written information remember to:

- use short words
- avoid long words and jargon
- explain technical terms
- use short sentences.

The material needs to be tailored to local needs. For example, when a large proportion of patients are illiterate, depicting written information through pictorial descriptions and illustrations will often be more effective. When using printed text^3^:

- use a minimum of font typeset 14, but 16–22 may be needed by some patients
- use black text on a white background
- use Arial or Verdana fonts
- avoid continuous capital letters and italics; use bold to make any emphasis
- use non-glossy paper.

If written information is handwritten, it must be black on white, in block capital letters, and equivalent to at least a printed size 16 (≥ 4mm).

### Patient teaching

When you teach patients a skill (e.g. instilling eye drops), it is important that you provide them with clear information and allow them to be active, rather than passive, in this teaching process. You should let the patient not only observe the skill, but also practise it under supervision, whilst giving them feedback. You should also give the patient the opportunity to ask questions.^4^

### Counselling

Counselling aims to help the patient discover solutions to his/her problems by exploring and clarifying ways of living that will increase his/her wellbeing. Unfortunately, this is often misunderstood. The object of counselling is not to inform the patient about his/her eye disease or to direct the patient towards a course of action. It is not prescriptive.

Counselling is a form of helping that is focused on the patient's needs, as perceived by the patient, and not on what others consider these needs to be. The counsellor does his/her best to listen to patients, working with them, to find the best ways to understand and resolve their problems. Counselling must take place in a private and confidential setting and counsellors should listen attentively to understand the patient's perspective. Counselling helps patients realise that there is a way for them to make a choice or change direction.^5^

Always remember that patients are individuals: despite having the same disease, different patients will have different needs.

There is evidence demonstrating many benefits of improved communication.^6^ Patients display better knowledge and are better able to recall information, they experience greater satisfaction with their health care, can give genuine and informed consent, and are able to cooperate better. As a result, they spend less time in hospital and experience a quicker recovery from illness and/or interventions. Consequently, they will relate their good experience to their community and this will create a better uptake of services.

## Staff accountability

It is important that all staff be accountable for the standard of care given. Improving the patient's experience is the responsibility of the whole eye care service delivery team.

Patients may be afraid to share their fears or complaints with doctors and nurses, thinking they are solely interested in treatment. They may be more comfortable sharing such comments with non-clinical staff; these personnel have a pivotal role in improving the patient's experience, as they often spend more time with patients. All personnel, therefore, should be alert and responsive to the patient's needs and give feedback to staff who can implement changes to improve the patient's experience.

Staff need to develop an awareness of their areas of competence, i.e. the skills, knowledge, and also the attitude (behaviour and belief systems) that they bring to their practice, as these will affect the way they treat patients.^7^

When acquiring experience, health care workers need to be self-aware and self-critical; they should seek supervision as they develop their own practice and offer supervision to junior colleagues. Continuing professional development (CPD), and being accountable for the standard of care given, results in relevant and appropriate delivery of care.

Finally, good time management is vital for improving the patient's experience. This encompasses prioritising, being systematic, and delegating where appropriate staff skills exist.^8^ Poor time management leads to a last minute rush, creates stress in staff and in patients, and leads to poor performance. The needs of patients and families must take priority and you should make sure they are promptly addressed.

## Dignity

Dignity is a basic human right. It is especially important to remember this in health care settings, where people feel more vulnerable. Staff must make every attempt to preserve a patient's dignity.

The care delivered to patients may be influenced by the quantity and quality of resources, but it must not be in any way restricted by their age, creed, culture, nationality, race, gender, disability, illness, political beliefs, education, or social and economic status.^9^

A patient's culture plays an important part in how he/she perceives dignity. It is important to show that you respect the patient's values. For example, ask the patient by what name or title they prefer to be called.

### Privacy

Ophthalmic patients require privacy. A patient interview or examination must happen away from spectators. You should take care to ensure confidentiality and store patient records securely.

Privacy for personal needs and hygiene is essential, with separate facilities for men and women. Very often, mixed wards are still unacceptable to individuals, though there is frequently no choice.

Accommodation for children should preferably be in a specifically dedicated and child-friendly space.

Patients in isolation, e.g. due to an infectious condition, may feel neglected or stigmatised. Staff should take time to interact with them outside of actual treatment procedures, to help increase a feeling of self-worth. When possible, it is helpful to provide separate accommodation for glaucoma and cataract patients, because these patients will experience different outcomes after surgery.

### Prioritising

In outpatient departments, and on operation lists, certain patients should be given priority over others (‘fast-tracked’): these are the very young, the elderly and infirm, those with acute or chronic general illness, and women (particularly those who are used to staying at home).

The patient is part of a family and community, and he/she may be supported by a parent, a sibling, a spouse or other carer. You should take time to address the needs and anxieties of carers too and, with the patient's consent, consult and include carers in any plans and preparation for the patient's discharge and ongoing care.

Staff must aim to be efficient and, most importantly, effective in supporting patients. They must be prepared to act as the patient's advocate and be assertive enough to challenge colleagues when they feel dignity is not given proper consideration.

## Environment

The ophthalmic health care setting is unique. Patients come from across the age spectrum and many have to cope with related health problems, as well as visual impairment. This poses a significant challenge, especially when eye care units are set up in under-resourced areas.

### Welcoming patients

Many patients travel long distances, and on arrival they will feel tired and vulnerable. The first person they meet will be very significant and will either raise or diminish their hopes and anxiety. The attitude of reception staff is crucial to the patient's feeling of wellbeing and self-worth.

### Cleanliness, safety, and comfort

Patients have a right to be cared for in a clean and safe environment. The housekeeping team are a vital part of the service and must be valued by clinical staff and senior managers.

The whole team is responsible for infection control, appropriate use of equipment, and reuse of materials. Health care settings must be places of safety and potential hazards must be noted.

**Figure:**
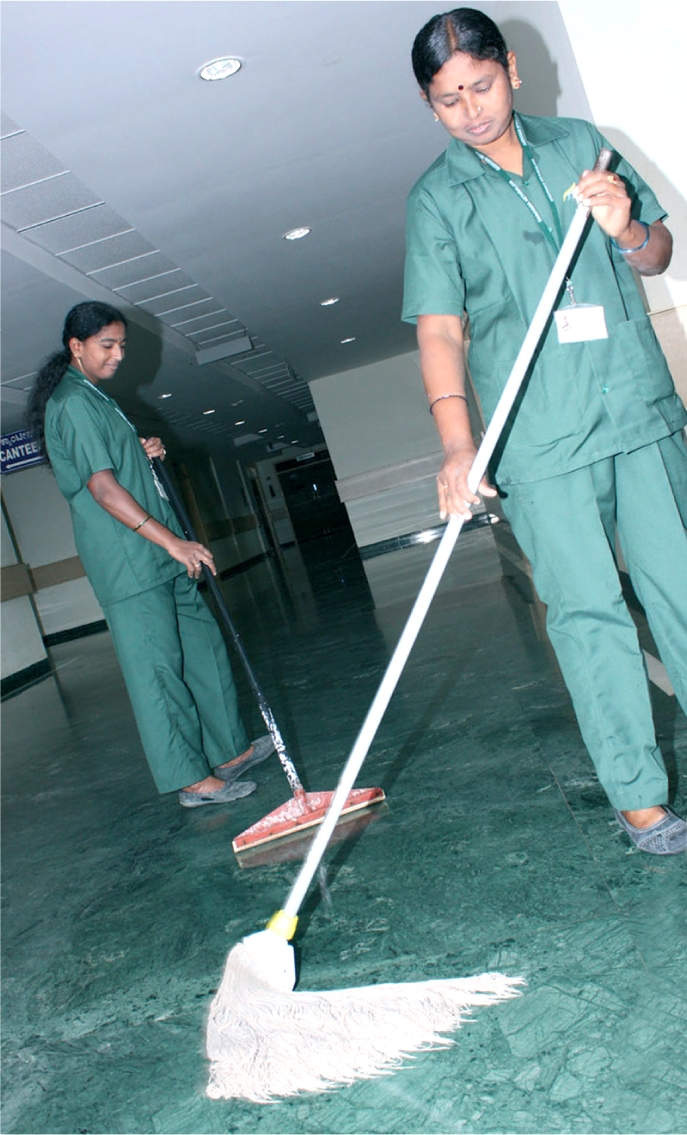
Ensuring that patients are cared for in a clean environment. INDIA

The maintenance department is responsible for equipment and furnishings requiring repair or replacement. In small hospitals where there are no separate housekeeping and maintenance teams, other staff should be given responsibility for these roles.

Good lighting is important for patients and for staff, in a speciality in which attention to detail is so vital. When the electrical supply is erratic, it is important to have backup generators and lamps, and to ensure that all staff know how to operate these.

### Accessibility

Patients may rely on notices and signs with written instructions. These must be accessible: they should be displayed in well-lit areas and in a colour and size that can be read easily.

Some patients will not have adequate vision to read even the best signage and may ask a staff member, for example: “Where do I collect my drops?”. It is not enough to point in the direction and reply: “It is down there on the right.” Patients expect staff to know how to assist them; however, staff are sometimes not aware of the needs and problems of patients with visual impairment. Staff should not be shy to ask patients about managing a situation specific to their personal needs.

Guidelines for eye health workers on assisting the blind and visually impaired should be discussed, techniques demonstrated, and instructions kept on display. Orientation days should be held for new staff to equip them with an understanding of the needs of the visually impaired.

There is only one correct way to guide a visually impaired patient and it can be described by the motto: “Don't pull me, walk with me.”^10^ Often, the wrong technique is used, causing difficulty and distress for the patient. Staff should always explain what is about to happen. Gentleness and patience are vital.

## Conclusion

The patient (and not the disease) must always be at the centre of ophthalmic services. Treating the patient as an individual will inevitably result in an improved service with increased outputs — but the outputs must never be the motivation for seeking such improvement.

**Glossary**

**Communication**: a two-way process, interaction with other(s), sharing of information and ideas.

**Continuing professional development (CPD**): instruction or opportunities for the purpose of updating and improving professional knowledge and skills.

**Counselling**: forum for confidential discussion and consultation, where problems are expressed by the patient. A counsellor must have listening skills, a sound knowledge of health, as well as experience and training.

**Dignity**: to treat someone with dignity is to make them feel important and valued in relation to others. When treated with dignity, a patient will feel confident, comfortable, and valued.

**Patient information (verbal and written**): conveys facts or instructions (e.g. the cost of an operation).

**Patient teaching**: provides knowledge and/or skills through discussion or demonstration (e.g. how to instil eye drops).

**Staff accountability**: the obligation of answering for the results or outcome of one's actions, as differentiated from responsibility (what we ought to do).
